# Religious diversity and public health: Lessons from COVID-19

**DOI:** 10.1371/journal.pone.0290107

**Published:** 2023-08-24

**Authors:** Lea Taragin-Zeller, Tamar Berenblum, Estefania Brasil, Yael Rozenblum, Ayelet Baram-Tsabari

**Affiliations:** 1 Federmann School of Public Policy and Governance and the Program in Cultural Studies, The Hebrew University of Jerusalem, Jerusalem, Israel; 2 The Haredi Institute for Public Affairs, Jerusalem, Israel; 3 Faculty of Education in Science and Technology, Technion – Israel Institute of Technology, Haifa, Israel; University of Malta Faculty of Health Sciences, MALTA

## Abstract

Scholars have identified a range of variables that predict public health compliance during COVID-19, including: psychological, institutional and situational variables as well as demographic characteristics, such as gender, location and age. In this paper, we argue that religious affiliation is also a clear predictor for compliance with public health guidelines. Based on a sample representative survey (N = 800) of Haredi Jews in Israel, we found that Haredi Jews mostly followed COVID-19 health regulations. Among the respondents who were non-compliant, however, we found large divergences which mostly reflected religious affiliation. While members of Lithuanian and Sephardi communities reported following guidelines, Hasidim, a more charismatic sub-group, were 12% and 14% more likely to flout public health guidelines than their Lithuanian and Sephardi counterparts, respectively. Despite this inner diversity, all Haredim were portrayed in Israeli media as one homogeneous group that was blamed for flouting public health guidelines and spreading COVID-19. Based on these findings, we argue for the importance of public health messaging that attends to diverse aspects of religious dogma, practice and observance by creating partnerships and sustainable relationships between different actors and stakeholders. In addition, we found that compliance was also shaped by knowledge about COVID-19 and public concern. Taking these findings together, health communication that acknowledges religious diversity while providing critical knowledge about the pandemic is key to developing and implementing community-focused interventions and public health programs. Practically, these insights help to improve pandemic governance as well as contributing theoretically to the study of public health relations and religion by highlighting how discourses around health vary and how differently positioned actors shape representations of responsiveness and health compliance.

## Introduction

COVID-19 was a painful reminder of how the health practices of ethnic, religious and migrant minorities often become the focus of heated political discourse during times of crisis. The rise in Anti-Asian hate crimes in the US is but one example of a long history of blaming “Others”, be it Irish Catholic immigrants during the Cholera outbreak or Italian immigrants blamed for spreading Polio [1–4]. Fueled by media, nationalist and public health discourse, minority groups around the globe are framed as being “irresponsible,” “dangerous” and potential hotspots of infection–reflecting broader claims of biological and social risks of transmission and contagion [5–9].

This global trend was also prevalent in Israel, where pictures of Haredim (a strictly Orthodox religious minority) defying public health guidelines featured prominently in the public sphere, constantly presented by media and politicians as a “risk” to the body politic [10–15]. Images of Haredim–demonstrating at rallies, praying in resistance, and defying Israeli soldiers–became a daily public spectacle during continuous lockdowns. In contrast to this public display of minority non-compliance, this paper goes beyond the threshold of Haredi homes to examine the varieties of Haredi public health compliance while highlighting inner group diversity. Haredim in Israel consist of three main sub-groups—Lithuanian, Sephardi and Hasidic Jews–who each behaved differently during COVID-19. Based on our survey (N = 800), we show that Hasidim, a more group-oriented stream of Haredi Judaism, were 12% and 14% more likely to flout public health guidelines than their Lithuanian and Sephardi counterparts, respectively (X^2^ = 35.4, p = .00, df = 2, N = 708).

Despite this diversity, all Haredim were portrayed in Israeli media as one homogeneous “black lump” and constantly blamed for flouting public health guidelines and spreading COVID-19 [12,14,16]. Not only did these gaps between reality and public imagery contribute to Haredim feeling like they were discriminated against [10], we argue that this gap can also obstruct public health compliance. In this particular case, disregarding inner group diversity contributes to stereotypes of a “dangerous” and “irresponsible” Haredi minority which can have psycho-social effects and also, in turn, effect adherence to public health guidelines [See: 3,17]. Charting these inner-group differences also allows us to push for a more nuanced understanding of the role religion actually plays in health decision-making. Recent studies reveal how the category of “religion” is often used to conflate a variety of factors that affect health decision-making [5]. This paper advances these growing understandings at the intersection of critical medical anthropology and religion, which often rely on qualitative data, to test these questions in a large scale quantitative study.

## Literature review

### Religion, health and COVID-19

As the pandemic reshaped the social world we live in, scholars turned to examine the ways different people followed social distance guidelines. Scholars have identified a range of variables that predict public health compliance during COVID-19, including: psychological, institutional and situational variables as well as demographic characteristics such as gender, geographical location and age [17–20]. Studies examining social distance guidelines in religious contexts have found that political affiliation is also key to understanding adherence to guidelines [21–23]. For example, Perry, Whitehead, and Grubbs found that in the US, people affiliated with the left were more likely to recommend precautions, while those on the (religious) right were more likely to disregard recommended precautions [22]. These findings resonate with Kahan’s argument that attitudes towards climate change tell us less about scientific reasoning and knowledge than about “latent cultural affiliations” [24], or the findings of a Pew Research Center survey that indicate that while more and more Americans are putting climate change as a national priority, democrats are much more concerned than their republican counterparts [25].

The implications of these studies are that people do not only encounter science and health-related decisions as members of particular groups, but they also seek out knowledge through pre-existing networks, utilizing prior acquaintances to help them understand new information. Recognizing the role that social context, including history, community, culture and religion play in shaping an individual’s ideas and worldviews, these findings resonate with established research from sociology, anthropology and history which has demonstrated how engagement with science is repeatedly shaped by social identity, historical context and power relations [26–29]. As Feinstein and Waddington put it: “People encounter scientific questions in social context—both as members of their social and cultural groups and with other members of those groups” [30]. Not only is the attempt to “filter out” these cultural frameworks unrealistic, it also assumes that these frameworks are detrimental to “proper” modes of rational decision-making, while, in reality they can be powerful and constructive resources for decision-making [31].

Well before the pandemic hit, religious minority groups were often perceived as “hard to reach” or as “non-compliant”. Even though these categories have been proved rather unhelpful, public health discourse and policies often use the term “religion” to account for differences in health-related actions, such as vaccine uptake [32]. Yet, recent scholarship shows that members of religious groups combine both scientific knowledge and socio-religious frameworks, which serve as “cultural and epistemological tunnels” [33,34]. In a recent study of COVID-19 health decision-making amid Haredi Jews [35], it was found that both health and religious justifications were used during sense-making and COVID-19-related decision-making. Whereas many respondents used general health-related justifications, many also utilized health-related justifications that were directly linked to religious language and culture, for example: ‘You shall take care exceedingly of your lives’ (Deuteronomy 4:15), suggesting that Haredi men and women have a particular vocabulary to express their justifications for following public guidelines.

In a similar vein, religious leaders are often perceived as key stakeholders in the medical decisions of religious individuals [32,36–38]. Within the emerging field of inclusive science communication, faith leaders are often portrayed as “trusted voices” within their communities who serve as sources of support, information and credibility [39–41]. Religious leaders can also identify ways to overcome particular community-based challenges, bridging understanding about the diverse ways health, healing and risk are conceived, especially in situations where health regulations can be perceived as undermining group-based priorities [see: 42–45].

### COVID-19 in Israel—Haredi varieties

Haredim (Ultra-Orthodox Jews) account for roughly 12.6% of Israel’s population [46]. Haredi men and women live according to the Hebrew Bible, which has been continuously interpreted through a large (and ever-growing) body of rabbinic literature and Jewish law. Haredi Jews can be easily identified by their relatively unified dress code (black hats and dark suits for men, and similarly colored ankle-length skirts, long sleeves, and head coverings or wigs for women). They are typically divided into three different sub-groups: Lithuanian *yeshiva*-based (Torah learning) communities; Hasidim, who place great emphasis on personal experience and more-charismatically oriented group worship; and Sephardi Haredim, who trace their origins to the Iberian peninsula, North Africa, and the Middle East. All Haredi sub-groups are often referred to as an enclave culture [47,48] with strict social and cultural boundaries and distinguished from other streams of Judaism: Progressive, Conservative, and religious-Zionist, by their avoidance of secular education and professional training [48–50].

The first case of COVID-19 in Israel was confirmed on 21 February 2020. From March 11, the Israeli government put forward an increasingly restrictive set of social distancing measures, culminating in a full lockdown by March 19. The number of infected cases rose rapidly during the last week of March, resulting in 6,092 confirmed cases by the beginning of April. During this initial stage of the pandemic, Haredi Jews were slower to adhere to social distancing guidelines than other groups in Israeli society [51,52]. Scholars have attributed this reluctance to various theological, cultural, and political causes. Some have blamed inner-communal media outlets for not reporting the dangers sufficiently, whereas, others have pointed to the ways social distancing disrupts the core of Jewish life which is based on religious obligations that can only be performed as a group, most notably collective prayer three times a day and religious study [53].

Others accused Haredi leaders for promoting non-compliance among their adherents, especially during the early stages of the pandemic. Shuki Friedman and Gabriel Even-Tsur [54] found that there was a clear link between the guidelines put forward by rabbinic leadership and their relationship to the state of Israel. Sephardi leaders, who often hold state-funded rabbinic positions, instructed their followers to abide by public health guidelines, as soon as these guidelines were issued. In contrast, Lithuanian and Hasidic leaders did not follow their example and waited a few weeks until they circulated similar guidelines. However, Lithuanian and Hasidic leaders only called on their followers to abide by public health deadlines, at the end of March 2020 as a response to the devastating hit on their communities. This delayed action contributed to the disproportionate effect of COVID-19 on the Israeli Haredi population, with major hotspots in Haredi neighborhoods and 40% to 60% of all coronavirus patients at four major hospitals, even though they make only 12% of Israel’s population [55,56].

As a response to this drastic blow, by the end of March 2020, all Haredi leadership put forward a clear message to follow public health guidelines, yet public instances of non-compliance were still clearly visible. Amidst this new context, this study was designed to examine the varieties of health decision-making among Haredim. This project advances recent interest in non-elite attitudes and science understandings [57,58] while analyzing the ways lay individuals incorporate Haredi sensibilities as part of their everyday decisions amid COVID-19. In doing so, our research also draws on growing scholarship pointing to the over-emphasis of religious leadership on health decision-making in religious contexts [38,59,60]. For example, Michal Raucher has shown that religious Jewish mothers are the primary carers of children and often responsible for managing health decision-making. She demonstrates how Haredi mothers “conceive” more authority after birthing four children, and navigate their own decision-making while disregarding both medical and religious authorities [38]. Similarly, Ben Kasstan has shown that mothers are important influencers in the context of vaccines, a fact which is often obscured when solely focusing on male religious authorities [32]. In this study we ask: How did Haredi men and women make health decisions during the pandemic? What types of justifications were utilized to support their decisions? How do demographic factors, such as age, education, gender and religious affiliation affect these decisions? And, what can these trends teach us about religious minorities and public health more broadly?

## Materials and methods

This paper is part of a large study exploring health decision-making in the context of COVID-19 among Haredi (ultra-Orthodox) Jews in Israel. As many do not have access to the internet, this paper is based on a telephone survey conducted by the Haredi market research firm “Eskaria”, which was administered to collect participants’ stances regarding COVID-19–related dilemmas. Information was gathered about education, age, gender, demography and religious affiliation and then participants were asked to respond to two COVID-19–related dilemmas that incorporated a potential conflict vis-à-vis social distancing guidelines. Each respondent was asked to report their solution to each dilemma in ways that best corresponded to their own attitudes and everyday decisions. Data was collected by “Eskaria” between 10–15 December 2020, at the height of the second wave of COVID-19, prior to a third national closure which was issued on 27 December 2020.

### Ethics

This study is a secondary analysis of this data, which was collected by "Eskaria” for the Haredi Institute for Public Affairs. The secondary analysis was waived by the IRB committee at the Technion: Israel Institute of Technology. Verbal consent was acquired by “Eskaria”.

#### Research tool

The questionnaire was constructed around real-life COVID-19 dilemmas to capture modes of health decision-making amidst the pandemic [See:^35^,^61^]. It also included measures of compliance, knowledge about COVID-19, and demographics.

### Content validity with experts

Content-related validity of the research tool was established non statistically using expert professional judgment, who addressed domain specification, content universe and sample, item development, item wording, and format. The questionnaire benefited from the constructive feedback of five members of the Haredi Institute for Public Affairs, which included both Haredi men and women as well as expert scholars of Haredi Judaism, all of whom are familiar with Haredi sensitivities. We also received feedback from three science communication and science education specialists, two of whom had a specific expertise in religion, with particular knowledge of Haredi society. We also received feedback from math, geography and science teachers to make sure that it resonated with general public literacy.

### Cognitive validity with target population

We gave five Haredim a draft of the questionnaire and asked them to fill it out. Given the tension related to vaccine hesitancy in Israel amid the pandemic, a few voiced concerns about the conclusions people might have from the dilemmas. In response to this concern, we made sure to phrase the questions in ways that don’t direct readers to make any decisions based on the questionnaire.

### Sample

Participants were recruited by the market research firm “Eskaria”, an online panel with particular expertise in conducting surveys among Hebrew speaking Haredim. The response rate was 18% and the entire sample included 800 participants. The limited response rate is not surprising given the relative length of this survey, the particular challenges of accessing Haredi publics for research purposes and the timing of the survey during the pandemic. This response rate might expose our data to a differential selectivity bias and limit the external generalizability of the findings. Nevertheless, our data offers important insights and data about inner group diversity that is often glossed over in academic research. Despite these difficulties, the final sample was representative of the general Haredi population regarding proportions of subgroups (Lithuanian, Hasidim, Sephardim).

In order to obtain an in-depth understanding of the varieties within ultra-Orthodox society, a random sample was chosen from Eskaria’s database that includes over 400,000 men and women in ultra-Orthodox society aged 18 and up. In terms of representativeness, the sample was similar to the general population in most categories. Almost all respondents (87%) were married, which reflects the high marriage rates in the Haredi population. Yet, the sample included more men (59%) than women (41%). While this balance was kept among Lithuanian and Sephardim, among Hasidim 82% of the respondents were male (Hasidic men 82% (N = 197), Hasidic women 18% (N = 44), Sephardi men 45% (N = 108), Sephardi women 55% (N = 132), Lithuanian men 48% (N = 109), Lithuanian women 52% (N = 120). Participants were also more educated than the Haredi average: 18% had completed a professional training course (compared with 29% in the general Haredi population), 23% had or were completing a BA degree (compared with 20% in the general Haredi population). The religious affiliation of respondents was also very similar to the general Haredi population, 30% (N = 240) belong to the Mizrahi sub-group, 29% (N = 229) to the Lithuanian community, 30% to the Hasidic community (N = 241), and 11% belongs to a variety of communities including Chabad, National-Haredi and Breslav (N = 90). Finally, we struggled to get older participants, and thus, most of our data is based on respondents between 21–44 (72%). Having said that, the Haredi population is relatively young: Haredim under the age of 17 are 53.5% of the Haredi population, compared to a national average of 28%; therefore the sample represents a very substantial part of the adult Haredi population (Haredi Institute for Public Affairs, 2020, based on data provided by the Central Bureau of Statistics).

### Limitations and future studies

Whether collecting data about actual compliance to COVID-19 or eliciting responses to hypothetical dilemmas, this data has been derived solely from the self-reports of the respondents. In addition, the survey was circulated during a time of crisis, which might have contributed to the relatively low response rate (discussed above) and might also reflect modes of decision-making that are specific to the pandemic context. Taken together, future studies which incorporate qualitative research methods conducted during less turbulent times will contribute to further understanding health-decision making among religious and ethnic minorities.

### Data analysis

#### Dependent variable

*COVID-19 decision-making*. Two dilemmas were designed to assess how Haredi men and women make COVID-related decisions amidst changing public health guidelines and recommendations. At the time, official guidelines allowed a maximum of ten people inside and twenty outside and schools were kept open. If exposed, there was a requirement to do whatever is necessary to stay home and test. Participants were asked how they would behave in social situations such as: Dilemma 1 (wedding): “Imagine your best friend scheduled a wedding for his son/daughter and the new guidelines now say that the wedding can only include twenty people outdoors. What would you recommend that they do?”. Dilemma 2 (elevator): “Your neighbor, who knew he had COVID, went into an elevator with you, without wearing a mask. As a consequence, you might have covid. Would you send your children to Talmud Torah (Jewish day school) in the next few days?”.

For each dilemma, we calculated the decision-making as follows:

Dilemma 1 (wedding)—Participants were presented with three options: 1. Postpone a wedding or fully adhere to wedding guidelines, 2. Recognize there is a problem and therefore have a wedding with a larger number of guests than allowed but still, less guests than one would usually have at a wedding, 3. Behave as if business is usual (and have a regular sized wedding). Based on these three options we created a new variable with two values, 1 = full compliance with guidelines (category one), 0 = partial or no compliance (categories 2 and 3).
Dilemma 2 (elevator)—Participants were presented with two options: (1) To send a possibly infected child to school or (2) Not to send a possibly infected child to school.

#### Independent variables

*Justification*. To analyze how each respondent explained their stance on each dilemma, we presented an open-end question to the participants, asking them to explain their choices. Based on content analysis we divided these justifications into ten variables: (1) *Reference to elderly population* (e.g. “We should take care of the elderly population); (2) *Health-related justifications*. This includes a reference to health, medical or scientific argumentations, e.g. “I can carry the virus even if I feel fine”; (3) *Public law or health authority recommendations* (e.g. “We should follow the guidelines and it is not a joke!”); (4) Reference to special actions that the respondents will do (e.g. “We will all wear masks so it will be fine”); (5) *Personal reasons* (“You only get married once”!); (6) *Lack of concern* (“It is not really that dangerous”); (7) *Religious justifications* (e.g. “Only god is really in charge of the world”); (8) *Public concern* (“e.g. I would never want to cause harm to another person”, (9) C*omparison between different situations* (“As long as we are going to work, we can go to a wedding” (10) *Other*.

*Compliance with MOH (Ministry of Health) guidelines*. To examine compliance with Ministry of Health guidelines (MOH), we created an index that examined the degree of compliance with individual guidelines. The index is made from a variety of statements, e.g. spatial distancing, hygiene, face-masks, and vaccination. For each statement, respondents were asked to rate the extent to which they would adhere to the guidelines between 1 (not strict at all) and 5 (very strict). The index was built as an average of four statements (N = 798, mean 3.7, SD = 0.95, range 1–5, Cronbach’s alpha = .69): “2 meters distance in public space” (N = 795, mean = 3.62, SD = 1.28), “mask wearing” (N = 796, mean = 4.34, SD = 1.06), “personal hygiene” (N = 793, mean = 3.82, SD = 1.36), “vaccination willingness” (N = 753, mean 2.36, standard deviation 1.13).

*Knowledge in the Context of Covid-19*. An open question about the efficacy of quarantine was used to assess knowledge about COVID-19. It was based on the response to the following question: “Any person who comes in contact with a verified patient is required to self-quarantine for 14 days from the moment of exposure, even though they had been out on the street for several days. Do you think isolation will help at this point?” Based on PISA’s definition of scientific explanation [62], we evaluated the application of scientific content knowledge to interpret and explain phenomena using the following categories: (1) respondent’s position regarding the efficacy of quarantine (intercoder reliability κ = .804), (2) correctness of the answer (intercoder reliability κ = .822), and (3) number and level of scientific concepts used. The concepts were characterized according to the author’s protocol [35], which is based on the inclusion of scientific concepts in Israeli science curricula for elementary, middle, and high school. Points were given only when respondents used scientific concepts correctly (e.g. “You can be contagious even if you do not have symptoms” versus “You are only contagious if you have symptoms”). One point was given for each elementary concept, two for each middle school–level concept, and three points for each high school–level concept. The scores for knowledge about COVID-19 ranged from 0 to 4, a higher score represents better knowledge (N = 800, mean = 2.04, SD = 1.15).

*Demand for knowledge*. Respondents were asked to rate how much more information about COVID-19 they would like to obtain in four different topics–math, vaccines, science and geography. The variable demand for knowledge was based on the sum of four variables: 1) Demand for math knowledge, 2) Demand for knowledge about vaccines, 3) Demand for knowledge in science, and 4) Demand for knowledge in geography (N = 800, mean = 6.3, SD = 4.3, range 0–13, 0 = no lack of knowledge, 1 = missing one item of information on a subject, 2 = missing two items of information, and so on).

*Trust in rabbinical leadership*. Respondents were asked to rate their degree of agreement between 1 (strongly disagree) and 5 (strongly agree) with the following statement: I have trust in rabbinical leadership (N = 787, mean = 4.70, SD = 0.79, range 1–5).

*Trust in Israeli government*. To examine trust in Israel’s government, we created an index based on the average of four questions: 1) I have faith in the Israeli government, 2) The government acts in an equal manner towards all citizens, 3) The Israeli government is making the right decisions for the residents of Israel with regard to the COVID-19 crisis, 4) Community restrictions following the COVID-19 crisis should be determined by the government. The variable ranks from 1–5: 1—Strongly disagree, and 5—Strongly agree (N = 800, mean = 2.7, SD = 1.01, Cronbach’s alpha = 0.77, range).

*Demographic variables*. The demographic variables included self-reports of gender, age, religious affiliation, economic status, community affiliation, all as multiple-choice items.

*Statistical analysis*. In order to assess the decision making and the justifications of the respondents we used Chi-squared and Fisher’s exact tests to determine the relationship between the variables. Logistic regression was conducted for each dilemma in order to explain the decision making factors.

## Results

In what follows, we showcase our main findings. To allow readers to capture the depth of each dilemma which presents a different type of conflict (personal decision vs. recommendation for friend; official guideline versus recommendation; potential conflict vs. actual conflict), each dilemma is presented separately and their results will be compared in the discussion section.

### Wedding dilemma

The first dilemma asked participants to state what advice they would give their best friend in a case where their son or daughter had planned a wedding during the pandemic. At that time, public health regulations capped inside public gatherings at twenty people and our participants were asked what they would recommend. In Judaism, marriage is valued highly and going to weddings especially among members of one’s extended family is very important. To answer this dilemma, participants were given three options—postpone the wedding, have the wedding with less guests than intended, or have a regular-sized wedding as usual.

**Fig 1:**
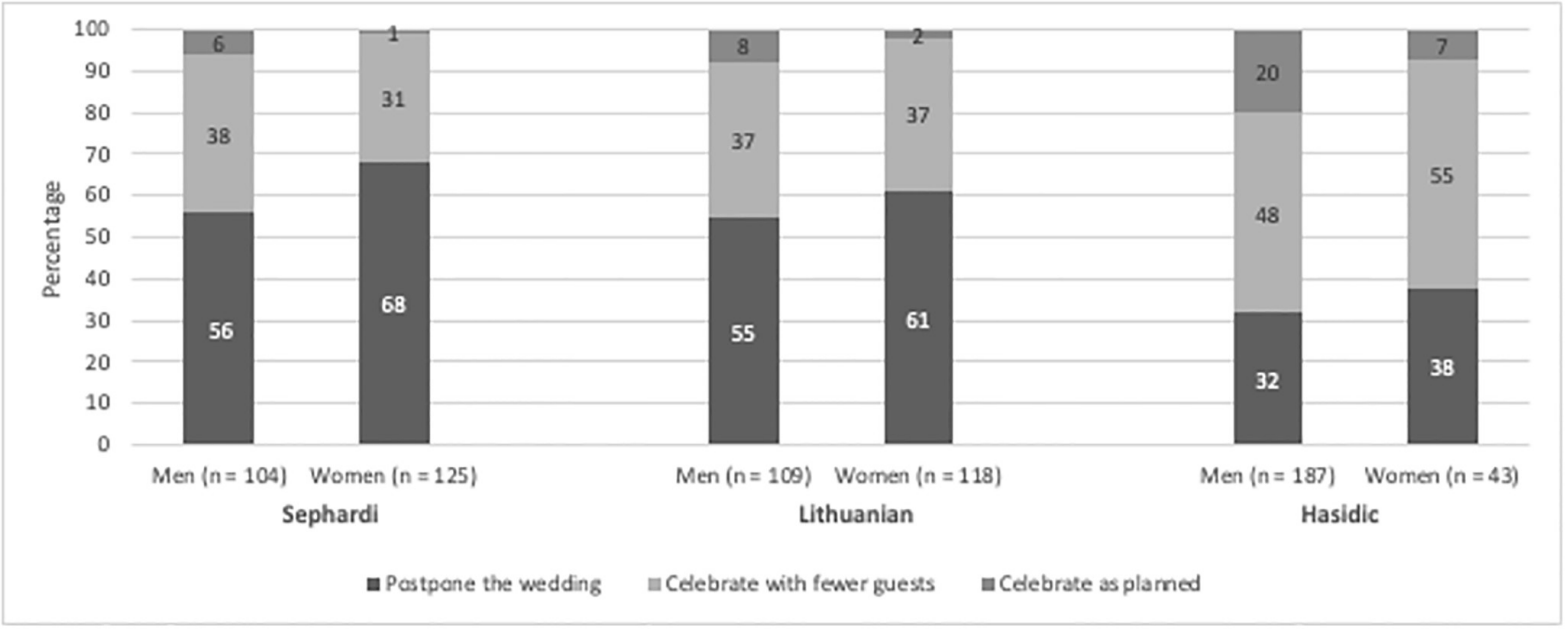
Responses of men and women within different Haredi sub-groups to wedding dilemma regarding social distance guidelines. Participants responses to the following dilemma: “Imagine your best friend scheduled a wedding for his son/daughter and the new guidelines now say that the wedding can only include twenty people outdoors. What would you recommend that they do?”.

51% of the participants reported that they take guidelines into consideration and fully comply with them. Yet, examining the responses of men and women within different Haredi sub-groups reveal big differences in adherence to social distance guidelines regarding weddings. Among Lithuanian and Sephardi haredim, most adhered to guidelines (58% and 63%, respectively), whereas only 33% of Hasidic Jews reported that they would follow guidelines (X^2^ = 60.23, p = .00, df = 4, N = 681). We also found that gender affected decision making as 61% of the women stated they would adhere to guidelines, compared to 45% of the men (X^2^ = 30.8, p = .00, df = 2, N = 769). When combining gender with religious affiliation, we found that Hasidic men are the group that reports the lowest level of compliance among haredi men (32%, X^2^ = 26.3, p = .00, df = 4, N = 393). In more detail (See Fig 1), Hasidic Haredi men reported that they would either fully adhere to guidelines (32%) or partially adhere to guidelines by celebrating with less participants (48%). Due to limitations with the number of observations in this question (described above), we conducted Fisher’s exact test for each sub-group to test for gender-related differences and did not find any significant differences within each sub-group (Sephardi, p = 0.07, N = 235; Lithuanian, p = 0.07, N = 221; Hasidic: p = 0.15, N = 225).

When asked to explain their decision, participants shared several justifications.

The respondents’ decision-making rationalizations were classified into 10 variables, in order of prevalence: (1) *Health-related justifications*—29.1% (2) *Public law or health authority recommendations*—25.3% (3) *Lack of concern* -12.7% (4) *Religious justifications*—12.5% (5) Reference to special actions that the respondents will do—6.5% (6) *Personal reasons—*5.1% (7) *Public concern*—4.2% (8) C*omparison between different situations*—2.3% (9) *Reference to high-risk populations public concern—*2.2% (10) *Other*—0.2%.

Within all communities, the most common justification was health-related. Health justifications came in different forms. Some respondents spoke about their own health “We must think about our health!” or “We need to stop the spreading of the virus!”. Others mentioned the importance of caring for special populations, especially the elderly. As Chava, a married, 33 year old Lithuanian woman put it: “We will not push off a wedding! We need to have the wedding as planned, but find a way to follow the guidelines and be careful”. When comparing different communities with regard to the justifications they used, we found that there is a significant difference between Hasidim and their Lithuanian and Sephardi counterparts. Reflecting this difference, Hasidim use less health justifications (21%) in comparison to the other communities (Sephardi 31% and Lithuanian 30%, X^2^ = 7.7, p = .02, df = 2, N = 710). While some Hasidim noted the importance of following guidelines, many responded that guidelines were politicized. For example, Baruch, a 33 year old Viznitz Hasid responded: “It is all politics”, and Avi, a 40 year old from Bet Shemesh explained: “We don’t have any trust in the state. They are full of nonsense!”, reflecting previous studies linking public health guidelines and state tensions [35,59], a point we will return to in the discussion section.

In order to examine the relationship between the various factors that predict decision-making in each dilemma, we conducted a regression logistic analysis. To do so, we use four different models to discern the effect of different groups of variables. The first column (model 1) presents the following demographic controls: age, gender, community affiliation and economic index. To check how education affects decision-making, the second column (model 2) takes the demographic controls (described previously) but also adds the following controls: demand for knowledge and COVID-19 knowledge. In the third column (model 3), we further included the following three controls: compliance with the MOH, trust in the Israeli government and trust in rabbinic leadership. Finally, in the fourth column (model 4) we also included: health-related and religious justifications.

In Table 1, we present the results for dilemma 1 (wedding). In the first column, gender has a positive significant relationship. Women are 37% more likely to follow guidelines than men. This effect wears off as we added the other types of specifications. Further, the main finding in all models is that religious affiliation is the most significant predictor. We found that both Sephardim and Lithuanians are much more likely to postpone a wedding than their Hasidic counterparts. Compared to Hasidim, Sephardim are three times more likely and Lithuanians are twice more likely to follow guidelines. Our findings also show that while the demand for knowledge shows a small effect at the start, this effect disappears after controlling for compliance and trust. However, we found that COVID-19-related knowledge shows a positive effect. As knowledge about COVID increases, the likelihood of recommending postponing the wedding increases by 32%. This result holds for the rest of the specifications, although the effect is reduced to 19–17% (columns 3 and 4, respectively). Further, compliance with the MOH was found to have the second strongest effect. Greater compliance with guidelines more than doubles the likelihood to recommend to postpone the wedding. When analyzing trust, the only significant one was trust in rabbinical leadership, which shows a negative relationship. Higher trust in rabbinic leadership decreases the likelihood to recommend to postpone the wedding by thirty percent. Also, health-related justifications present a positive relationship increasing the decision to recommend postponing the wedding by 72%. Finally, we used Shorrocks-Shapely value decomposition to demonstrate the relative contribution of each variable to the model. We found that compliance with MOH guidelines (52.7%), followed by Sephardi religious affiliation (12.3%), and health related justifications (8.33%) are the most influential variables for dilemma 1.

**Table 1:** *Dilemma 1*: *Wedding* (1 = Compliance with the guidelines).

	(1)	(2)	(3)	(4)
	odds ratio	odds ratio	odds ratio	odds ratio
Age	1.01	1.01	1.00	1.00
	(0.01)	(0.01)	(0.01)	(0.01)
Gender (Female = 1)	1.37*	1.27	1.22	1.22
	(0.24)	(0.23)	(0.24)	(0.24)
Sephardi (base of comparison: Hasidic)	2.95***	2.79***	2.80***	2.75***
	(0.63)	(0.60)	(0.67)	(0.66)
Lithuanian (base of comparison: Hasidic)	2.41***	2.25***	1.87***	1.83***
	(0.50)	(0.48)	(0.43)	(0.42)
Income (1 = Above or much above average)	1.31	1.25	1.11	1.11
	(0.24)	(0.23)	(0.23)	(0.23)
Demand for knowledge		1.04*	1.01	1.01
		(0.02)	(0.02)	(0.02)
Knowledge in the context of COVID-19		1.32***	1.19**	1.17*
		(0.10)	(0.10)	(0.10)
Compliance with MOH guidelines			2.55***	2.47***
			(0.31)	(0.31)
Trust in Israeli government			0.94	0.93
			(0.09)	(0.09)
Trust in rabbinic leadership			0.77**	0.77**
			(0.10)	(0.10)
Health-related justifications				1.72***
				(0.36)
Religious justifications				1.41
				(0.44)
Constant	0.33***	0.15***	0.05***	0.05***
	(0.09)	(0.05)	(0.04)	(0.04)
Observations	641	641	629	629
Likelihood ratio chi-square	49.82	69.61	140.5	147.8
Prob > chi2	0.00	0.00	0.00	0.00
Pseudo R-squared	0.0561	0.0784	0.161	0.169

Note: The table presents the relationship between the decision to recommend postponing a wedding and several explanatory variables. Standard errors are presented in brackets. Significance levels

*** p<0.01,

** p<0.05,

* p<0.1.

### Talmud Torah (Jewish day school) dilemma

The second dilemma asked participants what they would do if they were in an elevator with an unmasked neighbor, who they later found out had COVID-19. After posing this scenario, in which they were potentially exposed to COVID-19, we asked respondents whether they would send their children to school or not.

Our findings reveal that most of the participants (77%) reported that they consider public health guidelines and comply with them. Among those who reported they would send their child to school regardless of the elevator encounter, Hasidim were most likely to defy guidelines (31%), compared to their Lithuanian (23%) and Mizrahi counterparts (15%) (X^2^ = 16.84, p = .00, df = 2, N = 710). In this dilemma, gender was not found to be statistically significant (X^2^ = 1.87, p = 0.17, df = 1, N = 710).

**Fig 2:**
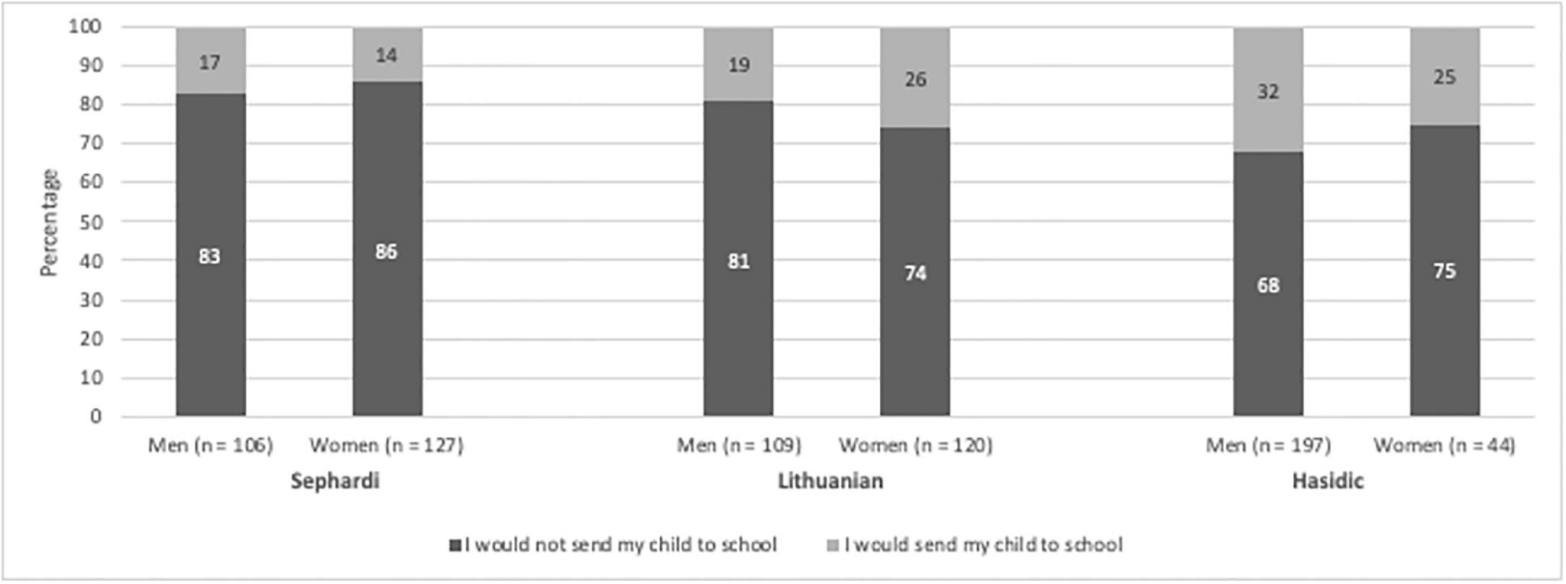
Responses of men and women within different Haredi sub-groups to Jewish day school dilemma regarding social distance guidelines. Participants responses to the following dilemma: “Your neighbor, who knew he had COVID, went into an elevator with you, without wearing a mask. As a consequence, you might have covid. Would you send your children to Talmud Torah (Jewish day school) in the next few days?”.

When asked to explain their decision, participants shared several justifications (See Fig 2). The respondents’ decision-making rationalizations were classified into 10 variables, in order of prevalence: (1) *Public concern*—32.1% (2) *Health-related justifications*—27.5% (3) *Lack of concern*—14.8% (4) *Public law or health authority recommendations*—13.9% (5) Reference to special actions that the respondents will do—5.8% (6) *Religious justifications*—2.2% (7) *Personal reasons—*1% (8) C*omparison between different situations*—0.6% (9) *Reference to high-risk populations public concern—*0% (10) *Other*—0%. In this dilemma, no significant differences were found between the different streams in using both religious and health justifications.

In contrast to the previous dilemma, in this dilemma the leading justification offered by participants was public concern. Public concern appeared in various forms: “We would not want to harm others”, “I would not want to be responsible for making other people sick”. As this dilemma was centered on sending ones’ child to school, the focus on harming others makes sense and was closely followed by health justifications, as Yoel, a twenty-eight year old Sephardi man from Bnei-Brak put it: “Health comes first!”.

Similarly to the first dilemma, we also examined the relationship between the various factors that predict decision-making in this second dilemma.

Table 2 presents the results for dilemma two. We found that age and gender did not have significant effects on the decision to send a possibly infected kid to school. Similarly to the first dilemma, religious affiliation is a central finding. Sephardi affiliation doubles the likelihood to keep a child at home, compared to Hasidim. Among Lithuanians, there is a positive effect in model one and two, which disappears in models (3) and (4). While knowledge has less of an effect in this dilemma, compliance with MOH reveals a positive strong effect. Those who comply with MOH guidelines are 86% more likely to keep their child home. We also found that both religious and health-related justification were significant: Health-related justifications are 27 times more likely to increase the likelihood to keep a child at home; and religious-related justifications decrease the likelihood to keep their child at home by seven times. Finally, we use Shorrocks-Shapely value decomposition to demonstrate the relative contribution of the variable to the model. We found that health-related justifications (48.5%), followed by compliance with MOH guidelines (28.7%) and Sephardi religious affiliation (6.2%), are the most influential variables for this dilemma.

**Table 2:** *Dilemma 2*: *Elevator* (1 = Compliance with the guidelines).

	(1)	(2)	(3)	(4)
	odds ratio	odds ratio	odds ratio	odds ratio
Age	0.99	0.99	0.98**	0.98*
	(0.01)	(0.01)	(0.01)	(0.01)
Gender (Female = 1)	1.04	0.99	0.89	0.91
	(0.22)	(0.21)	(0.20)	(0.21)
Sephardi (base of comparison: Hasidic)	2.49***	2.29***	2.03***	1.87**
	(0.62)	(0.57)	(0.54)	(0.52)
Lithuanian (base of comparison: Hasidic)	1.60**	1.47*	1.16	1.21
	(0.37)	(0.34)	(0.28)	(0.31)
Income (1 = above or much above average)	1.19	1.15	1.12	1.19
	(0.25)	(0.25)	(0.25)	(0.28)
Demand for knowledge		1.05**	1.02	1.02
		(0.02)	(0.02)	(0.03)
Knowledge in the context of COVID-19		1.22**	1.11	1.13
		(0.10)	(0.10)	(0.11)
Compliance with MOH guidelines			1.86***	1.86***
			(0.21)	(0.23)
Trust in Israeli government			0.99	0.94
			(0.11)	(0.11)
Trust in rabbinic leadership			0.95	1.00
			(0.13)	(0.15)
Health-related justifications				27.48***
				(19.89)
Religious justifications				0.14**
				(0.12)
Constant	2.78***	1.45	0.56	0.32
	(0.87)	(0.53)	(0.45)	(0.28)
Observations	669	669	657	657
Likelihood ratio chi-square	17.94	30.24	62.39	131
Prob > chi2	0.00	0.00	0.00	0.00
Pseudo R-squared	0.0247	0.0416	0.0872	0.183

Note: The table presents the relationship between the decision to send a possibly infected child to school and several explanatory variables. Standard errors are present in brackets. Significance levels

*** p<0.01,

** p<0.05,

* p<0.1.

## Discussion

Most participants reported that they follow MOH COVID-19 health regulations (with average of 3.7 out of 5 in the index). This finding resonates with a number of surveys demonstrating that Israelis were relatively compliant during COVID-19 [63,64]. In tandem with these findings, our survey showed that Haredi compliance was consistent in both dilemmas, which involved vastly different situations and presented different types of conflicts. Our findings also join a growing wave of scholarship that shows that Haredim followed public health guidelines, in sharp contrast to local and national media reports, which offered an overarching public depiction of Haredi non-compliance [13,65]. Among the respondents who were non-compliant, we found large divergences which largely reflected religious affiliation. While Lithuanian and Sephardi communities tended to follow guidelines, their Hasidic counterparts were much more likely to flout social distancing guidelines, especially Hasidic men. It is important to reiterate, however, that our findings are based on a relatively low response rate that it is exposed to differential selectivity and further studies are required to generalize these findings about inner Haredi diversity.

Notwithstanding, how can we make sense of this inner diversity? And, what are the broader ramifications of these findings? Hasidic Judaism, as noted above, is an umbrella term to include many sub-groups, such as: Belz, Bretslov, Lubavitch (Chabad), Sanz, Satmar, among others. Each Hasidic court is headed by a different Rebbe, called an *admor*, contributing to a decentralized infrastructure of rabbinic authority and distinct customs regarding prayer, dress, melodies and more. Many of the large Hasidic courts have deep links with their counterparts outside of Israel, especially in the US, which also influenced adherence to public health guidelines during the pandemic. Broadly speaking, Hasidic religiosity can be compared to other charismatic movements, which place a great emphasis on personal experience, emotions and spontaneity as well as ecstatic group worship.

A recent study examining Hasidic behavior during the pandemic examined the narratives of Breslov Hasidim who decided to continue their yearly pilgrimage to the Tomb of Rabbi Nachman of Breslov (1772–1810) in Ukraine, despite the closure of state borders. As the Ukrainian government announced the closure aimed at reducing contamination, many Breslovers attempted to travel before the closure to make their yearly pilgrimage. This resulted in thousands of Breslovers stranded in airports, land borders and even imprisoned in the days and weeks leading up to the annual pilgrimage on the Jewish new year. Anthropologist Rachel Feldman [66] argues that this choice was not a project of science denial but rather rooted in a conflict between state guidelines and religious practice. While Breslov Hasidim are considered one of the more ‘extreme’ Hasidic groups, Feldman’s analysis pushes us to think about the particular sensibilities of specific Jewish groups, especially the more charismatic and group-oriented ones, whose needs were not specifically targeted in public health guidelines [Also see 67]. As she notes, this chaotic pilgrimage is a painful mirror of what happens “when secular logics fail to contain and properly modify religious actors” [66, p.107].

Our findings resonate with this analysis. As Hasidic Jews followed public guidelines less than their Lithuanian and Sephardi counterparts, public health messaging that treats all sub-groups as one shade of black misses the inner group diversity, a misconception that might have vast ramifications. Black and colleagues [10] have shown that when Israeli authorities imposed closures of areas with high morbidity rates, which included much higher representation of ultra-Orthodox locations such as Bnei Brak (whose morbidity levels were much higher than those of the general population), the Israeli ultra-Orthodox population experienced a wave of frustration and expressed feelings of perceived discrimination in their sectorial press [See 68,69]. In addition, Folmer and colleagues have demonstrated how adherence to public health guidelines is also influenced by the behavior of other people in one’s community [17]. Following this logic, public portrayal of non-compliance creates a (misleading) impression that violations are normal among Haredim, which in turn has the potential to contribute to the growth of non-compliance from within. To be clear, our aim here is not to merely redirect blame. On the contrary, highlighting Haredi variety demonstrates the wide array of ideas and responses to the pandemic that must be accounted for in public health relations. Following Kasstan et al. [70], we argue that creating sustainable relationships between communal custodians and other positioned stakeholders can foster better understandings and collaboration in future events [71]. For example, a more targeted and diversified public health intervention, one that does not put all Haredim in the same (non-compliant) boat, would have been more conducive [Also see:, 6]. In fact, Israel’s Ministry of Health began a culturally specific science and health section to promote science and health communication on COVID-19 vaccination, which helped lower infection levels [52,72,73].

Finally, we also found that knowledge about COVID-19 (especially in the first dilemma) predicts compliance, even more than trust in religious leadership. Whereas many researchers note that Haredi Jews “refer to their rabbis for decision-making regarding medical procedures and screenings, so as to act in accordance with religious requirements” [37,74,75], our finding echoes recent scholarship that shows that decisions are not merely followed blindly, but rather negotiated in everyday life [76]. In contrast to this view, we found that health-related justifications and public concern were utilized by respondents who reported following public guidelines. In other words, health and religious frameworks were fused together in the decision-making process of respondents.

Taking these findings together, tailoring knowledge about the pandemic to specific group sensibilities might be the key to developing and implementing sustainable community-focused interventions, collaborations and public health programs [39]. For example, awareness of religious sensitivities and temporalities (e.g. New Year, as described above) must be incorporated in efforts to increase compliance amid diverse populations. Science and health communication that acknowledges the role of religious dogma, practice and observance while providing critical knowledge about the pandemic, can help us better prepare for future occurrences which will likely follow.
